# A characteristic signature of insulin-like growth factor (IGF) axis expression during osteogenic differentiation of human dental pulp cells (hDPCs): Potential co-ordinated regulation of IGF action

**DOI:** 10.1016/j.ghir.2018.07.003

**Published:** 2018

**Authors:** Hasanain Al-Khafaji, Pernille R. Noer, Hanna Alkharobi, Aishah Alhodhodi, Josephine Meade, Reem El-Gendy, Claus Oxvig, James Beattie

**Affiliations:** a Division of Oral Biology, Leeds School of Dentistry, Level 7 Wellcome Trust Brenner Building, University of Leeds, St James University Hospital, Leeds, United Kingdom; b Department of Oral Biology, Dental College, King AbdulAziz University, Jeddah, Saudi Arabia; c Department of Oral Pathology, Faculty of Dentistry, Suez Canal University, Ismailia, Egypt; d Department of Molecular Biology and Genetics, Aarhus University, Gustav Wieds Vej 10C, 8000 Aarhus C, Denmark

**Keywords:** IGF, insulin-like growth factor, IGFBP, insulin-like growth factor binding protein, PAPP-A, pregnancy-associated plasma protein-A, STC, stanniocalcin, ELISA, enzyme-linked immunosorbent assay, CM, conditioned medium, IP, immunoprecipitation, WB, Western blot, hDPC, human dental pulp cells

## Abstract

The IGF axis is represented by two growth factors (IGF1 and IGF2), two cognate cell surface receptors (IGF1R and IGF2R), six soluble high affinity IGF binding proteins (IGFBP1-6) and several IGFBP proteases. IGF1 and IGF2 are present at high concentrations in bone and play a crucial role in the maintenance and differentiation of both foetal and adult skeleton. In order to understand the role of the IGF axis in bone and other tissues it is necessary to profile the expression and activity of all genes in the axis together with the activity of relevant ancillary proteins (including IGFBP proteases). In the current report we used differentiating human dental pulp cells (hDPC) to examine the expression and activity of the IGF axis during osteogenic differentiation of these cells. We found that, with the exception of IGF1 and IGFBP1, all components of the IGF axis are expressed in hDPCs. IGFBP-4 is the most abundantly expressed IGFBP species at both mRNA and protein levels under both basal and osteogenic conditions. Although we found no difference in IGFBP-4 expression under osteogenic conditions, we report increased expression and activity of pregnancy associated plasma protein-A (PAPP-A - an IGFBP-4 proteinase) leading to increased IGFBP-4 proteolysis in differentiating cell cultures. Further to this we report increased expression of IGF-2 (an activator of PAPP-A), and decreased expression of stanniocalcin-2 (STC2- a recently discovered inhibitor of PAPP-A) under osteogenic conditions. We also demonstrate that STC2 and PAPP-A are able to form complexes in hDPC conditioned medium indicating the potential for regulation of IGFBP-4 proteolysis through this mechanism. We suggest that these changes in the expression and activity of the IGF axis may represent part of an osteogenic signature characteristic of differentiating hDPCs.

## Highlights

• Co-ordinated changes in PAPP-A, STC2 and IGF2 expression occur during osteogenic differentiation of human dental pulp cells (hDPCs). 2 This results in increased proteolysis of IGFBP-4 under osteogenic conditions 3. These changes in expression and activity provide a characteristic signature of IGF axis activity co-ordinated to enhance differentiation of hDPCs to a matrix mineralisation phenotype.

## 1 Introduction

The insulin-like growth factor (IGF) axis comprises two polypeptide growth factors (IGF1 and IGF2), two cell surface receptors (IGF1R and IGF2R) and six soluble high-affinity IGF binding proteins (IGFBP-1-6). The IGF axis plays a crucial role in the development and maintenance of the mineralised skeleton and disruption of the IGF1 or IGF1R gene compromises skeletal growth in rodents and humans [1, 2]. IGFs are the most abundant growth factors in bone tissue [3] and in addition to an anabolic role in mature bone, the IGF axis also regulates osteoblast and osteoclast differentiation in developing bone tissue, controlling the balance between bone accretion and resorption which occurs throughout life [[4], [5], [6]]. Accordingly, conditional knockout of IGF1 or IGF1R in osteoblasts or mature osteocytes compromises bone development and impairs osteogenic differentiation of mesenchymal stem cells [7, 8]. In bone and other tissues the activity of IGFs is regulated by the presence of high affinity IGFBPs [9] and we have previously demonstrated that IGFBP-2 and IGFBP-3 regulate exogenous IGF-1 stimulation of osteogenic differentiation in cultures of human dental pulp cells (hDPCs)·Therefore increased IGFBP-2 expression in these cells is associated with enhanced IGF-1 action while expression of the inhibitory IGFBP-3 is decreased during hDPC differentiation [10]. Although this may suggest a co-ordinated response of the IGF axis during osteogenic differentiation of hDPCs, differentiating osteoblasts also express IGFBP-4, -5 and -6 [[11], [12], [13]] and IGFBP-4 has been reported as the most abundant IGFBP in bone tissue [14]. To obtain a comprehensive account of IGF axis action in any cell culture system it is necessary to obtain full qualitative and quantitative data on IGF axis gene expression in order to be confident about how changes in gene expression and activity may impact on chosen biological endpoints. Surprisingly, with regard to quantitative aspects of IGF axis expression, very few studies have reported detailed profiles of gene and protein expression within cell culture models. In addition to this, IGFBPs are subject to post-translational modifications which have significant effects on their activity. Perhaps foremost amongst these is the proteolysis of IGFBPs into fragments with reduced IGF affinity. By this mechanism IGFBP proteolysis may regulate the partition of IGFs between high affinity IGFBPs and lower affinity cell surface receptors [15]. IGFBP proteases represent a broad range of enzyme activities with varying degrees of substrate specificity and it is important that the activity of these enzymes is acknowledged during investigations of IGF axis activity in selected cell and tissue culture models [16].

Dental tissues develop from oral ectoderm and neural crest derived mesenchyme and contain pluripotent stem cell populations which display a developmental potential similar to embryonic stem cells (ESCs) [17, 18]. Dental pulp tissue represents an accessible source of such cells and human dental pulp cells (hDPCs) have been extensively used in various cell differentiation studies [[19], [20], [21]]. Undifferentiated cells display a fibroblast-like morphology with associated high efficiency for adherent colony formation and high proliferative potential [22]. These properties suggest that adult dental tissues may provide a source of material (often discarded in the clinic) to provide multipotent cells for subsequent tissue engineering studies. To date the main use of such cells has been in hard tissue engineering programmes with a view to treatment of dental trauma. As such hDPCs have been differentiated down osteogenic/odontogenic lineages to generate appropriate 3-dimensional bone and or dentin structure [23, 24]. Although such strategies have met with some limited success they are hampered by an inadequate understanding of the effects of local growth factors on dental pulp cell differentiation. In the context of osteogenesis the IGF axis is known to play a crucial role in both the maintenance and differentiation of bone tissue (reviewed in [9] and see above). Similarly some recent studies have suggested a role for IGFs in regulating osteogenic/odontogenic differentiation of hDPCs [[25], [26], [27], [28]] although the majority of these studies have given little attention given to the role of IGFBPs in this process. Therefore in the current study we have used the hDPC culture model to report comprehensively on IGF axis expression and activity during the osteogenic differentiation of these cells. In addition we investigate the activity of the specific IGFBP-4 proteinase, pregnancy-associated plasma protein-A (PAPP-A), which is expressed as the sole IGFBP-4 protease by hDPCs, and the potential regulation of PAPP-A activity by stanniocalcin-2 (STC2) -a recently described proteinase inhibitor of PAPP-A. We describe a series of co-ordinated changes in IGF axis expression which we suggest may represent part of an “osteogenic signature” associated with differentiating hDPCs.

## 2 Materials

Tissue culture medium α-MEM, foetal bovine serum (FBS) and phosphate buffered saline (PBS) were from Lonza (Slough, UK). Penicillin/streptomycin (PS), l-glutamine, l-ascorbic acid and dexamethasone, were from Sigma (Dorset, UK). Tissue culture plastic was from Corning (Amsterdam, Netherlands). Taqman probes and primers were from ABI (see Supplementary Table 1). ELISA kits for assay of IGFBP-2 (#DGB200), IGFBP-3 (#DGB300), IGFBP-4 (#DY804), IGFBP-5 (#DY875), IGFBP-6 (#DY876), IGF1 (#DG100) and IGF2 (#DG200) were from R&D Systems (Abingdon, UK). RhIGFBP-4 (#804-GB), rhIGFBP-5 (#875-B5), rhIGF-1(#291-G1) and rhIGF-2 (#292-G2) were also from R&D Systems. Reagents for STC2 ELISA including recombinant (r) STC2 are described in [29]. Recombinant human (rh) PAPP-A was expressed and purified as described in [30]. PAPP-A antibodies for IP are described in [31]. PAPP-A (pico) ELISA kit (Ansh, TX, AL-101) was used for ELISA. Polyclonal rabbit anti-PAPP-A antibodies for WB following IP are described in [32]. Reagents for electrophoresis including 8–16% gradient polyacrylamide gels, ×4 SDS-PAGE sample buffer and molecular weight markers were from BioRad.

## 3 Methods

### 3.1 Tissue culture

Isolation, culture and osteogenic differentiation of DPCs have been described previously [10, 33]. These cells represent a heterogeneous population of stromal cells which contain a subset of cells which express the classical cell surface marker profile (CD90+/CD105+/CD146+/CD45-/CD31-) characteristic of pluripotent mesenchymal stem cells [[34], [35], [36]]. Cells were generally used between passage 3–5. Serum free medium was collected over a 24 h period from basal or differentiated cells. Typically media were collected at the end of a 7 day or 21 day differentiation period. Further details are provided in relevant figure legends. After freeze drying medium was reconstituted at ×5 concentration, aliquoted and stored at −80 °C prior to analysis.

### 3.2 ELISA

ELISA of IGFBP-2-6, IGF1, IGF 2 and STC2 and PAPP-A were carried out using capture based ELISA kits sourced as described above. CM samples were assayed at dilutions which fell within the standard curve range appropriate for the analyte in question. Unconditioned medium was used as a zero analyte control and to examine potential background reactivity. At the dilutions used no non-specific binding was evident. All other details of ELISA were conducted exactly according to manufacturer's instructions.

### 3.3 Western blot (WB)

CM samples were analysed by reduced SDS-PAGE following electrophoresis through 8–16% gradient polyacrylamide gels. Following electrophoresis (1 h; 100 V) gels were blotted to PVDF membranes using the semidry TurboBlot™ apparatus. Blots were blocked for 1 h in 0.1% TBS-Tween (TBS-T) containing 5% (w/v) BSA. Blots were subsequently hybridised with primary antibody (1:1000 in TBS-T for 1 h at RT or overnight at 4 °C). After ×3 washes in TBS-T blots were incubated with HRP conjugated secondary antibody for 1 h at RT, then washed ×3 in TBS-T and developed with SuperSignal™ enhanced chemiluminescent (ECL) reagent. Images were visualized, captured and semi-quantified using the GelDoc imaging system (BioRad). Estimation of the Mr of reactive bands was also achieved using GelDoc software along with concurrently run Mr standards.

### 3.4 qRT-PCR

TaqMan based real time quantification of target mRNA abundance was performed using an LC480 light cycler (Roche) as described previously [37]. GAPDH was used as housekeeping gene (HKG). Target genes are quantified as 2^−ΔCt^ relative to GAPDH and fold changes in gene expression basal v osteogenic are expressed as 2^−ΔΔCt^. Supplementary Table1 lists TaqMan assay identifiers.

### 3.5 Bioassay

IGF1 stimulation of ALP activity was examined over the range 0–100 nM exactly as described in detail previously [10]. When present IGFBP-4 and IGFBP-5 were co-incubated at 10 nM along with IGF1. Media (including IGF1 and IGFBPs) was changed on days 4, 7 and 10. Cultures were terminated at 14 days.

### 3.6 IGFBP proteolysis

Details of ^125^I-labelled IGFBP-4 proteolysis have been published previously [38]. Briefly, IGFBP-4 (10 nM) and IGF1 (100 nM) were incubated with 20 pM recombinant PAPP-A (rPAPP-A) or basal/osteogenic CM containing an equivalent concentration of PAPP-A (determined by ELISA see Fig. 2c). Inhibitory anti-PAPP-A antibody (1/41) or control IgG (both 20 nM) were present when required. Reactions were terminated after 6 h and levels of intact and fragmented IGFBP-4 determined by autoradiography as previously described [39].

### 3.7 Immunoprecipitation and WB

PAPP-A and STC2 immunoprecipitation was conducted exactly as described previously [29, 40, 41].No precipitation of target protein was seen with control IgG. Following elution of immune-precipitates proteins were separated by non-reducing 3–8% SDS-PAGE (1 h at 150 V) and transferred to PVDF membranes (Millipore). The blots were blocked for 5 min in 2% Tween 20 in ddH2O, then equilibrated in 50 mM Tris-HCl, 500 mM NaCl, 0.1% Tween 20, pH 9 (TST) and incubated overnight with primary antibodies (0.5–1.25 μg/ml) at room temperature. Antibodies were diluted in TST containing 2% skim milk (w/v). Blots were washed ×3 in TST, and incubated for 1 h at RT with secondary antibodies diluted in TST containing 2% skimmed milk (w/v). Blots were washed ×3 in TST and developed with enhanced chemiluminescence reagent (ECL Prime, GE Healthcare). Images were captured using an ImageQuant LAS 4000 instrument (GE Healthcare). Primary antibodies were rabbit polyclonal anti-PAPP-A at 1.25 μg/ml [40] and goat anti-STC1 at 0.5 μg/ml (R&D, AF2958). Secondary antibodies were Swine anti-rabbit IgG HRP (Dako, P0217) diluted 1:2000 and rabbit anti-goat IgG HRP (Dako, P0449) diluted 1:2000.

### 3.8 Statistics

**Fig. 1:**
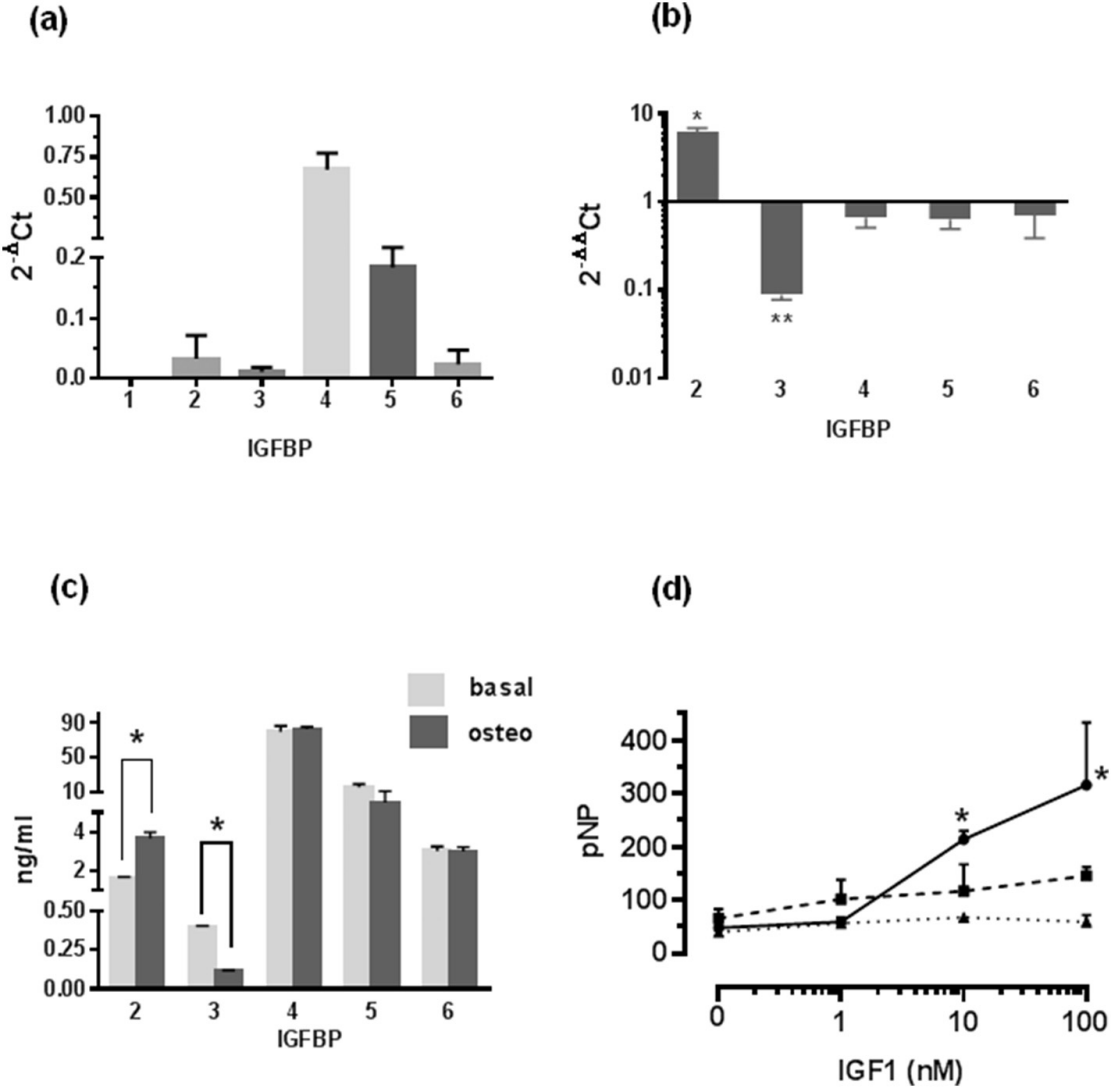
(a) qRT-PCR analysis of IGFBP expression in hDPCs cultured under basal conditions. Data are expressed as 2^-ΔCt^ relative to GAPDH and represent triplicate technical replicates of mRNA preparations from 3 different cell cultures. Mean ± SD (n = 3). IGFBP-1 was below detection limit of assay (Ct > 35). (b) Changes in IGFBP mRNA expression following osteogenic differentiation of hDPCs. Data are expressed as 2^-ΔΔCt^ osteogenic v basal and represent triplicate technical replicates of mRNA preparations from 3 different cell cultures. Data is presented as mean ± SD (n = 3) showing fold induction (>1) or decrease (<1) in IGFBP expression osteogenic v basal. *p < 0.005; **p < 0.0001. (c) IGFBP concentrations in basal and osteogenic hDPCs CM. hDPCs were treated under basal or osteogenic conditions for 7 days. CM was subsequently collected over a 24 h time period and IGFBP concentrations were determined by ELISA. Assays were performed as technical triplicates of 3 separate preparations of CM and are presented as ng/ml mean ± SD (n = 3); *p < 0.01. (d) Effect of IGFBP-4 or IGFBP-5 co-incubation on osteogenic activity of IGF1. IGF1 (filled circle) was present at 0, 1, 10 and 100 nM. IGFBP-4 (filled square) or IGFBP-5 (filled triangle) was present at 10 nM. Cells were cultured under osteogenic conditions and were terminated at 14 days. Lysates were prepared for determination of ALP activity using pNPP substrate as described in Materials & Methods. Triplicate technical replicates were performed on triplicate cultures and data are presented as mean ± SD (n = 3). Alkaline phosphatase (ALP) activity is expressed as nmol pNP formed/ug DNA. *p < 0.05 IGF1 alone v IGF1 + BP4/BP5. This experiment was repeated 3 times with similar results in each instance.

Differences between basal and osteogenic protein expression were analysed by Student's *t*-test and were considered significant at p < 0.05. For bioassay (Fig. 1d) one way ANOVA followed by Bonferroni's post-hoc test was used. Again differences were considered significant at p < 0.05.

## 4 Results

### 4.1 IGF axis expression

**Fig. 2:**
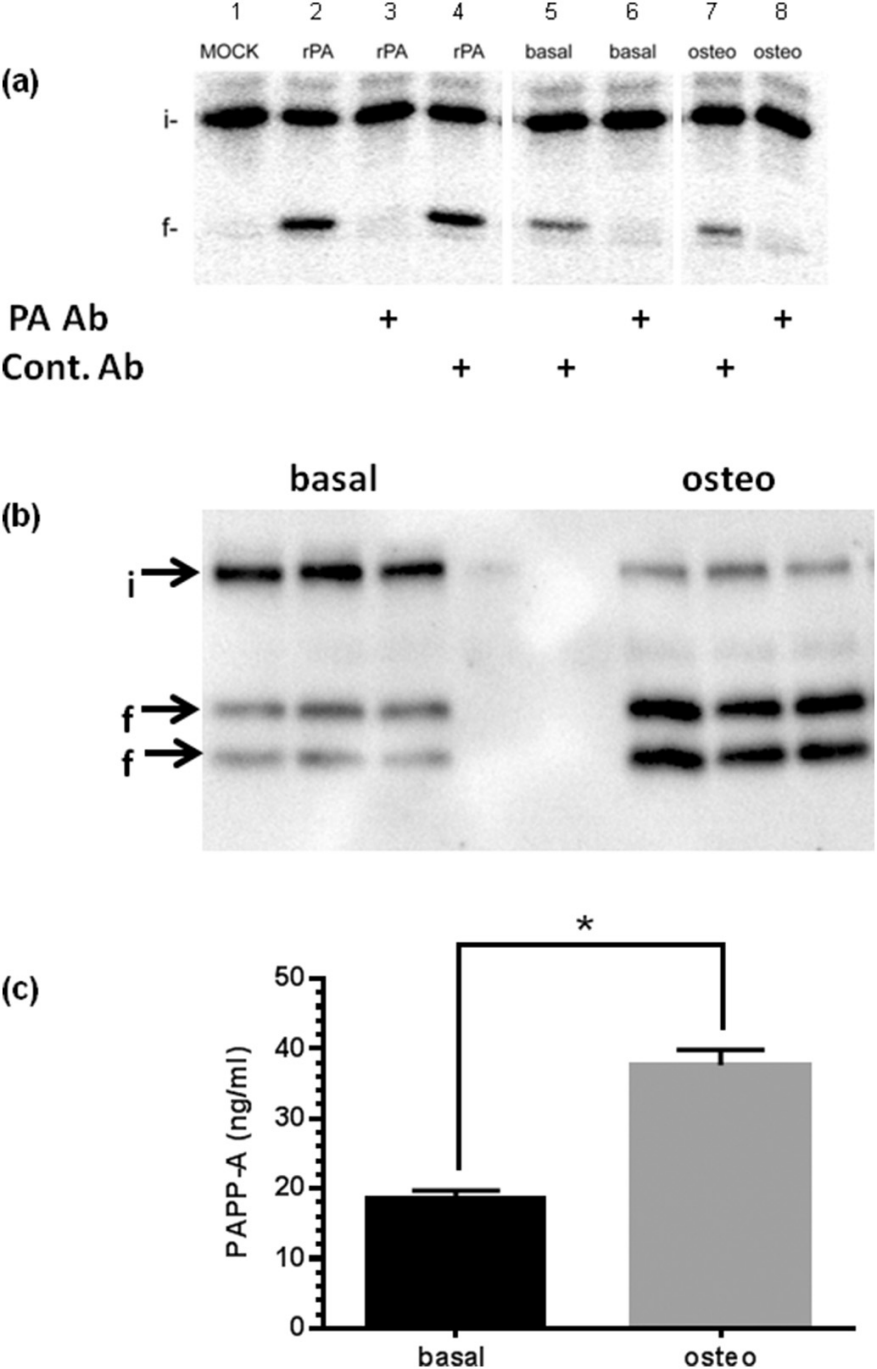
(a) Identification of IGFBP-4 proteolytic activity in hDPC CM. ^125^I-IGFBP-4 was incubated under cell free conditions with the following additions: Lane 1 control; lane 2 rPAPP-A only; lane 3 = rPAPP-A + inhibitory antibody (1/41 - PA Ab); lane 4 = rPAPP-A + control Ab; lane 5 = basal CM + control Ab; lane 6 = basal CM + inhibitory antibody; lane 7 = osteogenic CM + control Ab; lane 8 = osteogenic CM + inhibitory antibody. The location of intact (i) and fragmented (f) IGFBP-4 is indicated. See Methods for further details. (b) WB analysis of endogenous IGFBP-4 profile. Cells were treated under basal (B) or osteogenic (O) conditions for 7 days. CM was subsequently collected over a 24 h time period, freeze dried, reconstituted at ×5 concentration in dH_2_O then stored at −80 °C prior to analysis. Location of intact (i) and fragmented (f) IGFBP-4 are indicated (arrows). Triplicate lanes were run for both basal and osteogenic CM. The central two lanes contain unconditioned medium controls. This experiment was repeated on 5 occasions with similar results in each instance. (c) PAPP-A protein concentration in hDPC conditioned medium. hDPCs were treated under basal or osteogenic conditions for 7 days and serum free CM was subsequently collected over a 24 h time period. PAPP-A protein concentrations in CM were determined by ELISA. Assays were performed as technical triplicates of 3 separate preparations of CM and are presented as ng/ml mean ± SD (n = 3) *p < 0.01.

**Fig. 3:**
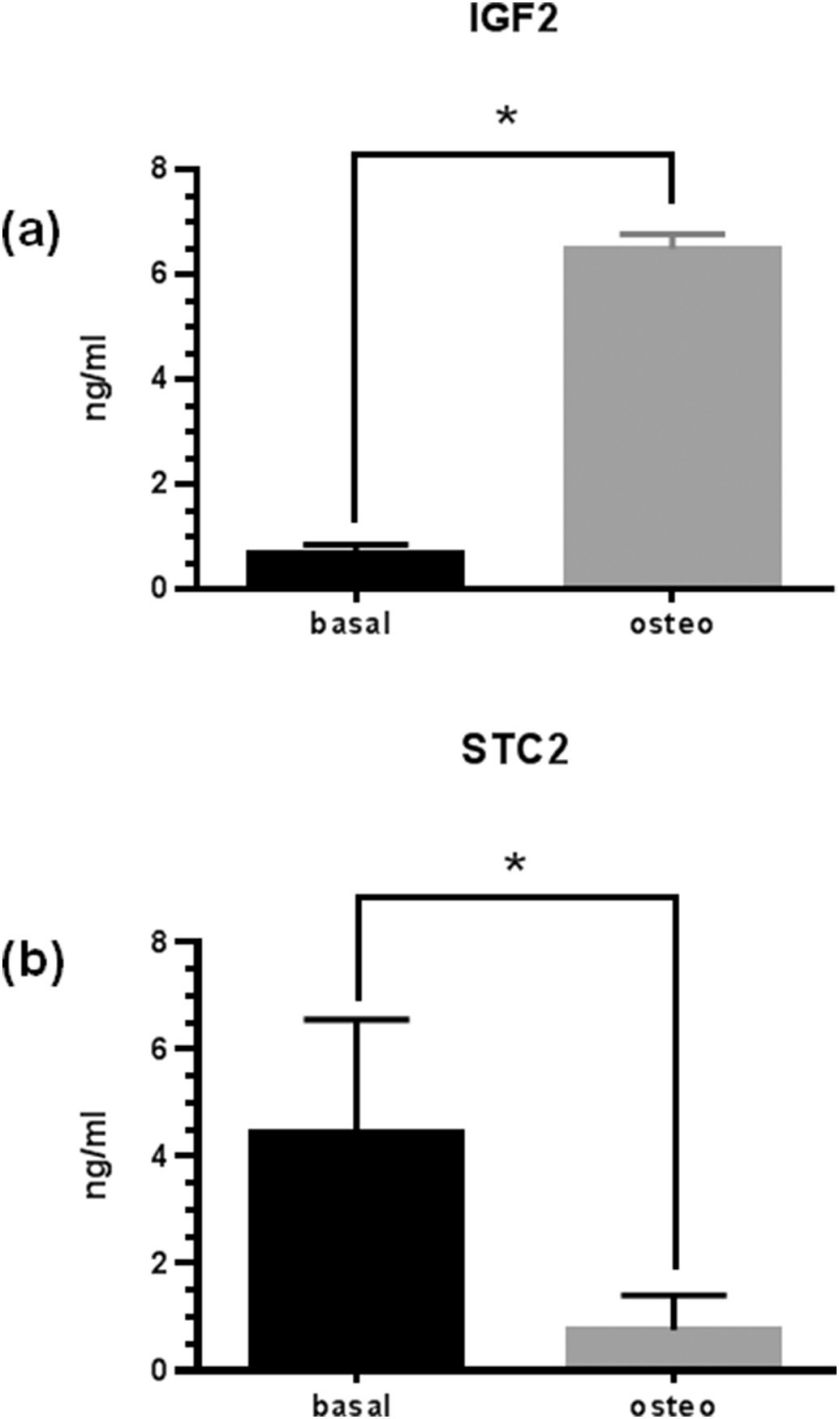
(a) IGF2 concentrations in basal and osteogenic CM. hDPCs were treated under basal or osteogenic conditions for 7 days and CM was subsequently collected over a 24 h time period. IGF2 concentrations were determined by ELISA. Assays were performed as technical triplicates of 3 separate preparations of CM and are presented as ng/ml mean ± SD (n = 3).*p < 0.01. (b) Stanniocalcin-2 (STC2) concentrations in basal or osteogenic CM. hDPCs were treated under basal or osteogenic conditions for 7 days and CM was subsequently collected over a 24 h time period. Assays were performed as technical triplicates of 3 separate preparations of CM and are presented as ng/ml mean ± SD (n = 3); *p < 0.01.

We found that under basal conditions, hDPCs express IGFBPs 2–6 (Fig. 1a), while IGFBP-1 mRNA was not detected. By qRT-PCR the order of mRNA abundance was BP4 > BP5 > BP2 = BP6 > BP3. Next we examined changes in IGFBP expression following osteogenic differentiation of hDPCs (Fig. 1b). We found reciprocal up and down regulation of IGFBP-2 and IGFBP-3 but no statistically significant change in IGFBP-4, -5 or -6 expression following osteogenic differentiation of hDPCs. This pattern was retained upon measurement of IGFBP protein levels in hDPC CM (Fig. 1c) where both absolute levels and changes in IGFBP protein concentrations following differentiation of hDPCs reflected the qRT-PCR data presented in Fig. 1a and b. IGFBP concentrations varied from 0.5 ng/ml (BP3) to 75 ng/ml (BP4) – Fig. 1c. This data indicates a close relationship between IGFBP mRNA and protein abundance and confirms and extends our previous observations [10]. IGF1 is widely reported to stimulate osteogenic activity both in vivo and in vitro [1], and we have previously shown that IGFBP-2 and -3 reciprocally regulate IGF1-induced osteogenic differentiation of hDPCs [10]. However, as IGFBP-4 and IGFBP-5 are by far the most abundant IGFBPs expressed by hDPCs (Fig. 1, Fig. 2, Fig. 3), we also examined the effect of these IGFBPs on IGF1-stimulated osteogenesis in hDPCs. Fig. 1d clearly shows that co-incubation of IGFBP-4 or IGFBP-5 (both 10 nM) with IGF1 (0-100 nM) inhibits the activity of IGF1 under osteogenic conditions.

### 4.2 IGFBP-4 proteolysis

IGFBP-4 and IGFBP-5 expression levels do not change under osteogenic conditions (Fig. 1b and c). However the activity of both of these IGFBPs can be modified post-translationally by proteolytic cleavage. In the case of IGFBP-4 the metzincin metalloproteinase pregnancy-associated plasma protein-A (PAPP-A) has been identified as the main IGFBP-4 proteinase expressed in fibroblast and osteoblast cultures [39]. To investigate whether proteolytic activity against IGFBP-4 was present in medium conditioned by hDPCs under basal or osteogenic conditions we used a cell free assay with ^125^I-IGFBP-4 as a substrate. IGFBP-4 proteolysis is dependent on the presence of IGF [42] and IGF1 was included in these assays (Methods section and see below). Fig. 2a shows that intact 28 kDa IGFBP-4 was proteolysed following incubation with medium conditioned by hDPCs grown under basal or osteogenic conditions with fragments co-migrating at 14 kDa. IGFBP-4 proteolysis was completely inhibited in the presence of an inhibitory antibody, indicating that PAPP-A is the only IGFBP-4 proteolytic activity expressed by hDPCs. IGFBP-4 has been described as an inhibitor of osteogenic differentiation due to high affinity binding of IGFs [43]. Proteolysis of IGFBP-4 reduces its affinity for IGFs allowing access of growth factors to cell surface receptors. As such IGFBP-4 proteolysis is viewed as a positive anabolic process in cells and tissues. In this light we examined whether endogenous IGFBP-4 proteolysis may differ between culture conditions. In Fig. 2b we show the results of a representative Western blot of conditioned medium collected over 24 h under basal or osteogenic conditions. It is clear from the data that the extent proteolysis of IGFBP-4 in osteogenic cultures (82.7 ± 3.3%) is greater than in basal cultures (38.2 ± 5.4%). We repeated these experiments with 5 different batches of hDPCs and obtained similar results in each instance. This suggested that PAPP-A enzyme activity and/or protein concentration may be higher in osteogenic medium compared to basally CM and that, at least over a 24 h time point, increased proteolysis of IGFBP-4 may occur. To address this issue, we directly assayed for PAPP-A protein in CM using ELISA. In Fig. 2c we show that PAPP-A concentrations are approximately 2-fold higher in osteogenic CM compared to basal CM in hDPC cultures treated for 1 wk.

### 4.3 IGF axis signature

**Fig. 4:**
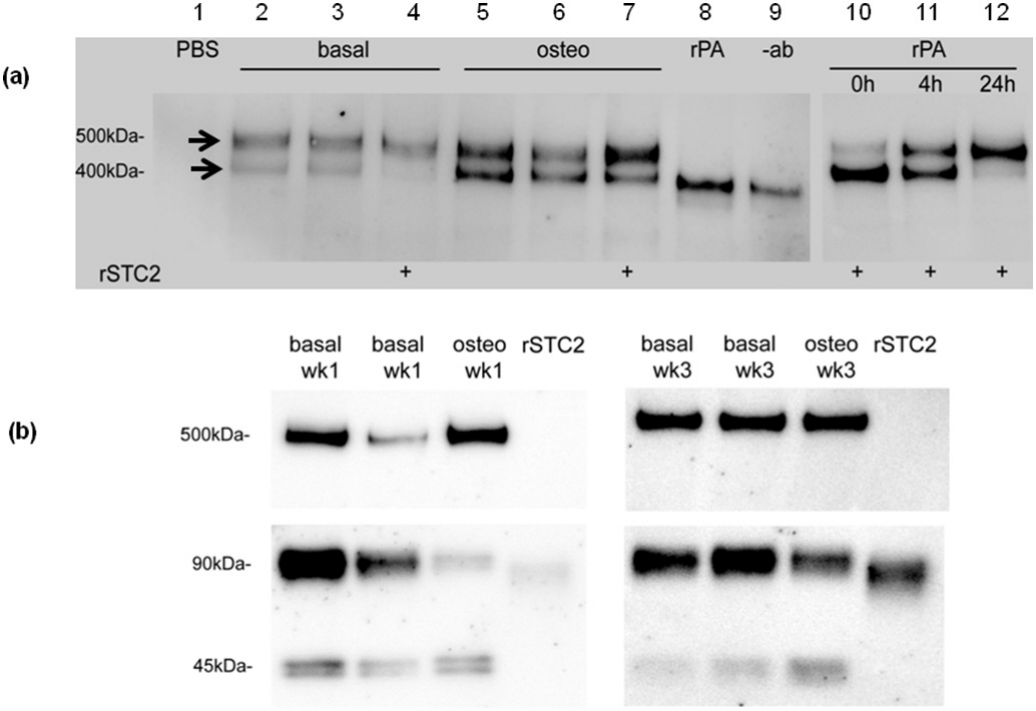
(a) Exogenous STC2 binds to endogenous PAPP-A in hDPC CM. Basal (lanes 2–4) or osteogenic (lanes 5–7) CM was incubated in the absence or in the presence (+) of exogenous rSTC2. PAPP-A and covalent PAPP-A: STC2 complexes were subsequently identified by IP and WB Western under non-reducing conditions as described in Methods. Lane 1- PBS control; lanes 2–4 basal CM in the absence (2 and 3) and presence (lane 4) of exogenous STC2; lanes 5–7 osteogenic CM in the absence (lanes 5 and 6) and presence (lanes 7) of exogenous STC2; lane 8 rPAPP-A alone; lane 9 no IP; lanes 10–12 time-dependent association of recombinant STC2 and rPAPP-A lane 10 = 0 h; lane 11 = 4 h; lane 12 = 24 h. Conditions of IP/WB as for lanes 1–9. (b) STC2 complexes in hDPC CM. STC2 was immunoprecipitated from basal (lanes 1,2,5 and 6) or osteogenic (lanes 3 and 7) hDPC medium conditioned after 1 wk (lanes 1–3) or 3 wk (lanes 5–7) Immune complexes were analysed by WB using a specific anti-STC2 Mab. The central portion of the blot has been removed and the location of STC2 monomer (~45 kDa), dimer (~90 kDa) or STC2: PAPP-A complex (>500 kDa) is indicated Control lanes contain rSTC2 (lanes 4 and 8). Gels were run under non-reducing conditions. See Methods for further details.

As indicated above, proteolysis of IGFBP-4 is IGF dependent. Therefore our data showing endogenous proteolysis of IGFBP-4 suggests that bioactive IGF(s) are present in CM. qRT-PCR data indicated that IGF2 but not IGF1 was expressed by hDPCs (data not shown). ELISA of CM confirmed the presence of IGF2 in hDPC cultures (Fig. 3a). Furthermore, under osteogenic conditions, IGF2 concentrations are increased over almost 10-fold (0.69 ± 0.17 v 6.49 ± 0.28 ng/ml). Stanniocalcin-2 (STC2) is a recently described highly potent inhibitor of PAPP-A activity [29]. In order to investigate whether differences in STC2 concentrations may be associated with the differences in PAPP-A activity between basal and osteogenic hDPC cultures, we determined STC2 concentrations in CM. Fig. 3b shows an almost 8-fold decrease in inhibitory STC2 concentrations osteogenic compared to basal CM (4.4 ± 2.1 v 0.77 ± 0.64 ng/ml) – Fig. 3b. STC2 forms a high-affinity covalent complex with PAPP-A which results in the inhibition of PAPP-A activity. Such complexes can be visualized by non-reducing SDS-PAGE as a high molecular weight band (>500 kDa), representing a 2:2 complex between PAPP-A and STC2 [29]. To investigate whether endogenous PAPP-A in hDPC CM can form such complexes with STC2, we carried out the experiments described in Fig. 4a. Analysis of both basal (lanes 2 and 3) and osteogenic (lanes 5 and 6) CM following sequential IP/WB for PAPP-A indicated the presence of both uncomplexed PAPP-A (~400 kDa) and higher molecular weight complexes representing PAPP-A: STC2 complexes (>500 kDa). The presence of such complexes was confirmed in both basal (lane 4) and osteogenic (lane 7) CM by the addition of exogenous STC2. Under these conditions in basal CM uncomplexed PAPP-A is almost completely removed into the slower migrating PAPP-A: STC2 complex. In osteogenic CM while there remains some uncomplexed PAPP-A following addition of exogenous STC2 there is evidence of increased intensity of the slower migrating complex (compare lanes 5, 6 v lane7). These differences between basal and osteogenic CM may reflect the higher concentrations of PAPP-A in osteogenic CM (Fig. 2c). Importantly the data presented in Fig. 4a is consistent with the observation of increased PAPP-A activity evident in osteogenic CM (Fig. 2b and c) and is significant because in the absence of a covalent complex STC2 is not inhibitory. Time-dependent association of rPAPP-A and rSTC2 is shown as positive control in lanes 10–12 of Fig. 4a. Finally, to confirm the presence of STC2 in complexes from hDPC CM, the experiments described in Fig. 4b were conducted. IP followed by non-reducing WB of STC2 in both basal (lanes 1, 2, 5 and 6) and osteogenic (lanes 3 and 7) CM show STC2 present as a high Mr complex (>500 kDa) as well as free STC2 dimer and monomer migrating at 90 and 45 kDa respectively. Although the data for the basal 1 wk CM is variable (lanes 1 and 2) there is evidence that in osteogenic CM at both 1 and 3 wk there is less free STC2 present as the 90 kDa dimer (compare lane 3 v lane 1 + 2 and lane 7 v lane 5 + 6). This data is consistent with lower concentrations of STC2 in osteogenic compared to basal CM (Fig. 3b). Taken as a whole our data suggest that in hDPC cells PAPP-A activity may be regulated by formation of PAPP-A: STC2 complexes. Molar concentrations of selected metabolites described above are presented in Supplementary Table 2.

## 5 Discussion

We have quantified IGFBP mRNA and protein expression in hDPCs and shown that with the exception of IGFBP-1 hDPCs express each of the well characterised high affinity IGFBPs. IGFBP-4 is the most abundant IGFBP expressed under both basal and osteogenic conditions. Surprisingly very few studies have reported quantitative data for all IGFBP species expressed in a given cell culture system. This is rather surprising given that a complete picture of IGFBP profile (qualitative and quantitative) is required for the interpretation of both IGF-dependent and IGF-independent effects of IGFBPs. In general terms IGFBPs are thought to be expressed at higher concentrations than IGFs in osteoblast and fibroblast cultures [44]. This is the case in our hDPC cultures under both basal and osteogenic conditions where global IGFBP concentration is approximately 4 nM and IGF2 is present at 0.1 nM. IGF1 mRNA and protein were undetectable in our hDPC cultures. Therefore under basal conditions this represents an IGFBP: IGF ratio of 40 and suggests that the majority of IGF2 in hDPC CM is bound to IGFBPs. Importantly, under osteogenic conditions, IGF2 concentrations increase 10-fold to approximately 1 nM reducing the IGFBP: IGF ratio to 4 (Fig. 3a). This may have important consequences for osteogenic differentiation of hDPCs through direct stimulatory effects of IGF-2 on osteogenic differentiation [45] but also through increased proteolysis of IGFBP-4 (see below). Although we did not detect IGF1 mRNA or protein in hDPC cultures, IGF1 is known to promote the osteogenic differentiation of hDPCs [25, 27]. In this regard it is important to note that dental pulp is a well vascularised tissue and IGF1 may therefore access DPCs through the systemic circulation or via paracrine expression in neighbouring cells. Therefore the extent of proteolysis of high affinity IGFBPs (including IGFBP-4) in the local environment remains relevant in considerations of local IGF activity. Our finding of higher expression of IGF2 than IGF1 in hDPCs has also been reported previously in osteoblast cultures [46, 47] and in DPCs [45].

We have recently shown that IGFBP-2 enhanced and IGFBP-3 inhibited the pro-osteogenic effects of IGF1 in hDPCs. This was co-ordinated with increased and decreased expression of IGFBP-2 and IGFBP-3, respectively, during differentiation of these cells [10]. The expression of IGFBP-4 and IGFBP-5 did not change during hDPC differentiation (Fig. 1b and c), but as these IGFBPs are 10–100 fold more abundant than IGFBP-2 or IGFBP-3 (Fig. 1a and c), we investigated the effect of IGFBP-4 and IGFBP-5 on the osteogenic activity of IGF1. Fig. 1d shows that both IGFBPs at equimolar concentrations inhibit the pro-osteogenic action of growth factor. For IGFBP-4, this inhibitory effect is in agreement with most in vitro studies in cultured osteoblasts [48, 49]. For IGFBP-5, both inhibitory [[50], [51], [52], [53]] and stimulatory [[54], [55], [56], [57]] effects on osteoblast activity have been described. We found no evidence of enhancement of IGF1 activity by exogenous IGFBP-5 added to hDPC cultures. In addition, neither IGFBP-4 nor IGFBP-5 stimulated ALP-activity independently of IGF1. Interestingly the inhibitory effect of IGFBP-4 and -5 was not overcome by increasing concentrations of IGF1. Although we have no ready explanation for this, we would emphasise that the affinity of IGFBPs for IGFs is 10-fold higher that of IGF1R and it may be that IGFBP-4 and-5 can concentrate IGFs within the abundant extracellular matrix secreted by hDPCs. Investigations into this phenomenon are continuing in our laboratory.

**Supplementary Fig. 1S:**
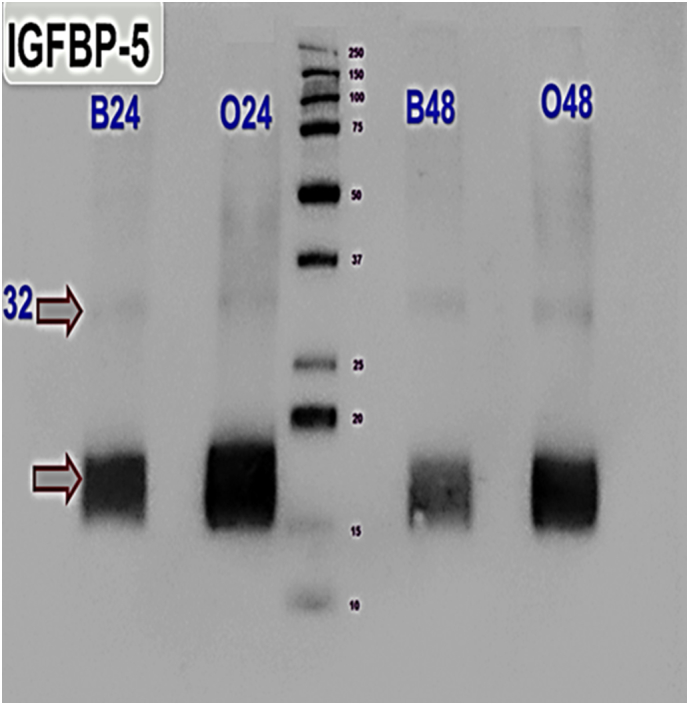
WB analysis of IGFBP-5 in medium conditioned by hDPCs for 24 or 48 h. Expected location of intact IGFBP-5 at 32 kDa is identified in addition to a broad band of reactivity between the 15 and 20 kDa Mr markers.

Although we show that IGFBP-4 concentrations are not altered following osteogenic differentiation of hDPCs, it is well established that the activity of IGFBP-4 in fibroblast and osteoblast cultures can be modified by IGF-dependent proteolysis [58, 59]. We therefore examined whether there was any difference in IGFBP-4 proteolysis between basal and osteogenic hDPC cultures. Initial experiments in cell free assays suggested that most, if not all IGFBP-4 proteolysis was due to the activity of PAPP-A present in CM (Fig. 2a). To investigate proteolysis of endogenous IGFBP-4, we conducted WB analysis of hDPC CM. We found that IGFBP-4 was proteolysed into fragments which migrated at 18 and 14 kDa in reducing SDS-PAGE (Fig. 2b). This is similar to previously described endogenous IGFBP-4 proteolysis in osteoblast cultures [60]. However, in medium conditioned for 24 h under basal conditions, we repeatedly observed that a higher proportion of intact IGFBP-4 was present compared to CM from osteogenic cultures (Fig. 2b). As IGFBP-4 protein concentrations are equivalent by ELISA between basal and osteogenic media (Fig. 1c and Supplementary Table 2), this suggests increased IGFBP-4 proteolysis under osteogenic conditions. Note that the IGFBP-4 ELISA detects both intact and fragmented IGFBP-4 species and that the difference between co-migration of IGFBP-4 fragments in cell free assay compared with the resolvable IGFBP-4 fragments evident during endogenous IGFBP-4 proteolysis (compare Fig. 2a and b) is explained by the use of a tagged recombinant IGFBP-4 species for cell free assay. We also performed WBs for endogenous IGFBP-5 in hDPC CM (Supplementary Fig. 1S). In this instance, we found that little intact 32 kDa IGFBP-5 was present in either basal or osteogenic hDPC CM with most protein running as a broad band between 15 and 20 KDa. This may represent the proteolysis of IGFBP-5 by several proteinases known to act on this IGFBP each with different cleavage sites [61, 62].

As PAPP-A proteolysis of IGFBP-4 is IGF dependent, this suggests that IGFs are present endogenously in hDPC CM and actually bound to this IGFBP and we confirmed the expression of IGF2 in hDPC cultures (Fig. 3a). Indeed IGF2 is a more potent activator of IGFBP-4 proteolysis than IGF1 [38] and significantly the concentration of IGF2 was approximately 10-fold higher in osteogenic vs. basal medium (Fig. 3a and Supplementary Table 2). This increased IGF2 concentration under osteogenic conditions would be expected to increase the fraction of IGFBPs (including IGFBP-4) associated with IGF2. This would in turn increase substrate availability (IGFBP-4:IGF2 complexes) for PAPP-A and may contribute towards increased proteolysis of IGFBP-4 under osteogenic conditions. Importantly increased local IGFBP proteolysis may enhance the action of systemically derived IGF1 which reaches dental pulp tissue. In this instance therefore IGF2 and IGF1 are seen to act in concert to promote cell differentiation. To examine the hypothesis of increased PAPP-A activity in osteogenic medium more directly, we assayed PAPP-A protein levels in basal and osteogenic CM by ELISA (Fig. 2c). This indicated increased PAPP-A protein in osteogenic medium vs. basal medium. In 1 wk. cultures PAPP-A concentrations were increased approximately 2-fold under osteogenic conditions. These findings are consistent with a pattern of gene expression and/or activity which promotes IGF activity during osteogenic differentiation of hDPCs. It is also worth comment that these concentrations of PAPP-A represent a rather high endogenous enzyme-to-substrate ratios (between 1:2 and 1:10) and may partly explain the extensive proteolysis of endogenous IGFBP-4 evident under both basal and osteogenic conditions. Also worthy of note in our experiments, no difference was apparent in PAPP-A mRNA expression between basal and osteogenic hDPC cultures in either 1 wk or 3 wk cultures (data not shown). This suggests that increased PAPP-A protein concentration in osteogenic CM is the result of post-transcriptional or post-translational mechanisms. Although we have no ready explanation for this at present, PAPP-A is known to associate with cell membranes [63] and we are investigating the possibility that increased release of membrane associated PAPP-A protein may occur under osteogenic conditions. The activity of the IGFBP-4:PAPP-A axis has been recently reviewed [64].

Recently, PAPP-A enzyme activity has been shown to be inhibited by both STC1 and STC2 [29, 65]. To determine whether PAPP-A activity may be regulated by either of the STCs, we initially examined the expression of STC1 and STC2 using qRT-PCR. We found that STC2 mRNA was over 100-fold more abundant than STC1 in hDPC cultures (data not shown). Further to this, ELISA of CM indicated that STC2 levels in osteogenic medium were almost 6-fold lower than those in basal medium (Fig. 3b).This lower concentration of inhibitory STC2 in osteogenic compared to basal medium is also consistent with a hypothesis of co-ordinated pro-osteogenic changes in IGF axis activity during osteogenic differentiation of hDPC cultures. Comparing the concentrations of PAPP-A and STC2 under basal or osteogenic conditions indicates that the PAPP-A: STC2 ratio is close to 1 under basal conditions and 10 under osteogenic conditions (Supplementary Table 2). Although the stoichiometry of the covalent PAPP-A: STC2 complex is 2:2, we find that even under basal conditions, IGFBP-4 is proteolysed. This indicates that an active fraction of PAPP-A exists in basal CM and suggests that covalent complex formation between PAPP-A and STC2 might be a regulated process. In order to confirm that endogenous PAPP-A present in hDPC CM is able to form a complex with STC2, we conducted the experiments described in Fig. 4a and b. IP and WB of basal and osteogenic CM with anti-PAPP-A antibodies showed the presence of a 500 and 400 kDa complex. The former of these represents endogenous formation of PAPP-A: STC2 complexes and the latter uncomplexed PAPP-A. Addition of rSTC2 to basal CM decreased the intensity of the free PAPP-A species suggesting increased association of PAPP-A with added STC2. This effect was also seen in osteogenic CM along with an increase in intensity of the more slowly migrating PAPP-A: STC2 complex. This data suggests that in hDPC CM endogenous PAPP-A and STC2 can form complexes and that PAPP-A activity may be regulated by this interaction. Finally to confirm the presence of endogenous STC2 in high-molecular weight complexes in hDPC CM we carried out IP/WB experiments with anti-STC2 antibodies as described in Fig. 4b. Under these conditions a substantial proportion of STC2 is present in the form of high-molecular weight complexes in both basal (lanes 1, 2, 6, 7) and osteogenic (lanes 3 and 8) conditions – Mr ~500 kDa. This was seen in cultures grown for both 1 wk and 3 wk under basal or osteogenic conditions. This provides further evidence that endogenous PAPP-A:STC2 complexes exist in medium conditioned by hDPC cultures under both basal and osteogenic conditions and represents a mechanism by which PAPP-A and therefore IGFBP-4 proteolysis may be regulated in these culture conditions.

In conclusion, we have shown that co-ordinated transcriptional and post-transcriptional changes occur in the IGF axis which may facilitate the osteogenic action of IGFs during differentiation of these cells. These include increased IGF2 expression; increased PAPP-A expression; decreased STC2 expression; the presence of STC2: PAPP-A heterodimers presenting a potential regulatory action for PAPP-A. These changes culminate in increased IGFBP-4 proteolysis (the most abundant IGFBP expressed by hDPCs), potentially allowing increased access of IGFs to cell surface IGF1R which is abundantly expressed in these cells. The use of dental pulp derived stem cells for bone tissue engineering has been the subject of a recent systematic review [66] and the role of the IGF axis in this process in such cells is also the subject of some primary literature [24, 67, 68]. As appropriate growth factor signalling cues are required for successful tissue engineering strategies, we believe our data may be relevant for the further development and use of hDPCs in such strategies for example where bone tissue formation and maintenance may be compromised (e.g. osteoporosis) and may provide new molecular targets for anti-osteopenic therapeutic approaches.

The following are the supplementary data related to this article.

Supplementary Table 1S Taqman assay identifiers see https://www.thermofisher.com for further details. Table 2S Concentrations of selected metabolites in CM of hDPCs. hDPCs were grown for 7 days under basal or osteogenic conditions and CM subsequently collected over a 24 h time period. Data are expressed as pM and represent mean ± SD (n = 3). Image 1

## Conflict of interest

The authors declare no conflict of interests.
