# Supplementary material for: A characteristic signature of insulin-like growth factor (IGF) axis expression during osteogenic differentiation of human dental pulp cells (hDPCs): Potential co-ordinated regulation of IGF action
Source: Growth Horm IGF Res. 2018 Oct-Dec;42-43:14–21. doi: 10.1016/j.ghir.2018.07.003 (PMC6259625; doi:10.1016/j.ghir.2018.07.003)
Supplement: Supplementary file 1 — Supplementary Table 1S Taqman assay identifiers see https://www.thermofisher.com for further details. Table 2S Concentrations of selected metabolites in CM of hDPCs. hDPCs were grown for 7 days under basal or osteogenic conditions and CM subsequently collected over a 24 h time period. Data are expressed as pM and represent mean ± SD (n = 3). [file mmc1.docx]

**Supplementary Table 1**

| ***Gene Name*** | ***TaqMan®Gene expression***  ***assay identifier*** |
| --- | --- |
| ***GAPDH*** | **Hs99999905_m1** |
| ***ALPL*** | **Hs01029144_m1** |
| ***OCN*** | **Hs00609452_g1** |
| ***Runx2*** | **Hs00231692_m1** |
| ***IGF1*** | **Hs01547656_m1** |
| ***IGF2*** | **Hs04188276_m1** |
| ***IGF1R*** | **Hs00609566_m1** |
| ***IGF2 R*** | **Hs00974474_m1** |
| ***IGFBP 1*** | **Hs00236877_m1** |
| ***IGFBP 2*** | **Hs01040719_m1** |
| ***IGFBP 3*** | **Hs00426289_m1** |
| ***IGFBP4*** | **Hs01057900_m1** |
| ***IGFBP 5*** | **Hs00181213_m1** |
| ***IGFBP 6*** | **Hs00181853_m1** |
| ***PAPP-A*** | **Hs01032307_m1** |
| ***STC1*** | **Hs00174970_ m1** |
| ***STC2*** | **Hs01063215 _m1** |

**Supplementary Table 2**

| **Analyte** | **Basal (pM)** | **Osteo (pM)** |
| --- | --- | --- |
| **IGF2** | **92 ± 22** | **863 ± 37** |
| **IGFBP-4** | **3072 ± 257** | **3180 ± 111** |
| **IGFBP-5** | **542 ± 136** | **346 ± 48** |
| **PAPP-A** | **47 ± 2.5** | **92 ± 4** |
| **STC2** | **49 ± 24** | **8.5 ± 7.2** |
